# Supplementary material for: Empirical methods for controlling false positives and estimating confidence in ChIP-Seq peaks
Source: BMC Bioinformatics. 2008 Dec 5;9:523. doi: 10.1186/1471-2105-9-523 (PMC2628906; doi:10.1186/1471-2105-9-523)
Supplement: Additional File 1 — A variety of html documents from the USeq web site detailing the available applications, their best usage, output file type descriptions, command line menus, etc. [file 1471-2105-9-523-S1.zip › USeqUserGuides/JavaDoc.zip!/JavaDoc/bioroot/antibody/class-use/AntibodyPrep.html]

Uses of Class bioroot.antibody.AntibodyPrep


|  |  |  |
| --- | --- | --- |
| **Overview** \| **Package** \| **Class** \| **Use** \| **Tree** \| **Deprecated** \| **Index** \| **Help** | |  |
| PREV   NEXT | **FRAMES**    **NO FRAMES**     **All Classes** |  |


---


## **Uses of Class bioroot.antibody.AntibodyPrep**

| Packages that use AntibodyPrep | |
| --- | --- |
| **bioroot.antibody** |  |

Uses of AntibodyPrep in bioroot.antibody

| Methods in bioroot.antibody that return AntibodyPrep | |
| --- | --- |
| `AntibodyPrep[]` | `AntibodyBean.getAntibodyPreps(DBUtil bioRoot)` |

| Methods in bioroot.antibody with parameters of type AntibodyPrep | |
| --- | --- |
| `static int` | `AntibodySpreadSheet.addUseDilutionBlankLines(boolean useDilutionsPresent, java.lang.String[] items, AntibodyPrep prep, int k, DBUtil bioRoot)`             Adds "" to items based on the number of use dilutions present in the Antibody Prep bean. |
| `static void` | `AntibodyBase.getAntibodyPrepForm(AntibodyPrep a, DBUtil bioRoot, java.lang.StringBuffer b, int index)` |
| `static void` | `AntibodyBase.getAntibodyPrepReport(AntibodyPrep a, DBUtil bioRoot, java.lang.StringBuffer b, int index, boolean commonForm)` |
| `static void` | `AntibodyBase.getUseDilutionForms(AntibodyPrep a, DBUtil bioRoot, java.lang.StringBuffer b, int apbIndex)`             Pumps out useDilution forms, should always have one with each Prep |
| `static void` | `AntibodyBase.getUseDilutionReports(AntibodyPrep a, DBUtil bioRoot, java.lang.StringBuffer b, int apbIndex, boolean commonForm)`             Pumps out useDilution reports |
| `static void` | `NewAntibody.loadUseDilutions(java.util.HashMap nameValue, AntibodyPrep apb, int antibodyPrepIndex, javax.servlet.http.HttpServletRequest request)` |
| `void` | `AntibodyBean.setAntibodyPreps(AntibodyPrep[] antibodyPrepBeans)` |
| `static boolean` | `AntibodyModify.updateCommonAccessFields(AntibodyPrep[] preps, DBUtil bioRoot)`             Just update the commonly accessible fields in each antibodyPrep as well as the useDilutions. |

---


|  |  |  |
| --- | --- | --- |
| **Overview** \| **Package** \| **Class** \| **Use** \| **Tree** \| **Deprecated** \| **Index** \| **Help** | |  |
| PREV   NEXT | **FRAMES**    **NO FRAMES**     **All Classes** |  |


---
